# Motherhood

**Published:** 1914-07

**Authors:** 


### Motherhood.

In the intervals between the suffering which she had endured since midnight, she again went over their dreams. They wanted a little girl—she would be so much company to them, and they would name her for both of their mothers. She could almost feel the little body in her arms as she rocked it to sleep. She even sang in her mind the songs she would croon to it. And she hoped that the nurse would get the right dress to put on it, the one she had made all with her own hands—the first one she ever had made. If anything should happen to her, there was the best little dress, all trimmed with lace and lovely ribbon, to put on it. And they had even talked of how it would learn to talk and to walk and to call them "mother" and "daddy." Ah, they soon would know whether their dream was to be fulfilled—or if—no, no, she wouldn't think of that!

The hours passed by, slow of torture. The pains had become so frequent and were so cruel that she no longer thought of those beautiful dreams: she only thought of how could she bear another such ordeal. The voices of the nurse and the physician seemed far, far away, and they did not seem to relate to herself at all. The perturbed husband by her bedside, his face pale, leaned over and whispered something to the physician. Then, "The doctor says that it will be just a few minutes more, dear, and that you are doing splendidly," he spoke encouragingly, as he bent down over her and stroked her pallid cheeks. But she could only smile at him, for that dreadful, maddening pain had come on again.

She closed her eyes and, every nerve taut, she said to herself: "How can I stand it? Oh, I can not bear it. They told me not to catch my breath—I'll try to hold out a moment longer, but can I? If only I could relax." Then, "Oh! Oh! O my God," she unwittingly cried out aloud. And then, harken—

"Ba-ah, ba-ah," weakly, faintly, breaking the stillness of the chamber.

"O! It's come," she whispered—and such a look of holy joy flitted over her face as it is permitted us to see but seldom in a life's time.

"And it's a fine boy, too," the doctor chortled, as he held up a little pink body and gave it a few smart slaps upon the back.

Not a shade of disappointment passed over her face. She smiled and closed her eyes. In a few minutes she again opened them and, glancing at her husband, who still sat by her bed and with his head resting on his hand, said, "I don't care a bit that it's a boy, do you?"

He, a big strong man, yet, his body shook with sobs and tears welled from his eyes when he raised his head. The strain had been too much for him.

"Why dear," she said, "you shouldn't cry now, everything is all right, the baby is all right." But tears fell on her hand as he leaned over and reverently kissed it.

"You had better go and see how he is," she murmured.

He soon returned to her, a smile all over his face: "Why, he weighs more than ten pounds and is kicking so that the nurse can hardly dress him." And again she smiled.

When, in a short while, all had been put to order and she lay there in the stillness while the blessed sleep of exhaustion crept over her, she wondered how it was she deserved such happiness. She forgot that she had suffered until every fiber of her body pained so that she scarce dared breathe, and felt so weak that it cost an effort merely to lift her hand. Wasn't everything all right? Wasn't the baby all right? And she smiled in the darkness.—*American Journal of Clinical Medicine.*

